# Expression of SCF splice variants in human melanocytes and melanoma cell lines: potential prognostic implications

**DOI:** 10.1054/bjoc.1999.1076

**Published:** 2000-03-21

**Authors:** P Welker, D Schadendorf, M Artuc, J Grabbe, B M Henz

**Affiliations:** Department of Dermatology, Charité, Humboldt University, Augustenburger-Platz 1, Berlin, D 13344, Germany; Medical University of Lübeck and Clinical Cooperation Unit for Dermato-Oncology (DKFZ), Mannheim University Clinic, Germany

**Keywords:** SCF splice variants, c-Kit, melanocytes, melanoma

## Abstract

Stem cell factor (SCF), the ligand for c-Kit, is known to regulate developmental and functional processes of haematopoietic stem cells, mast cells and melanocytes. Two different splice variants form predominantly soluble (sSCF or SCF-1) and in addition some membrane-bound SCF (mSCF or SCF-2). In order to explore the prognostic significance of these molecules in melanoma, total SCF, SCF splice variants and c-Kit expression were studied in normal skin melanocytes and in 11 different melanoma cell lines, using reverse transcription polymerase chain reaction, immunocytochemistry and enzyme-linked immunosorbent assay. Nine of the 11 melanoma cell lines expressed SCF-1 mRNA, only two of them SCF-2, and these two also SCF-1. Coexpression of both SCF-1 and c-Kit was noted in five cell lines, and only one cell line as well as normal melanocytes expressed both SCF-1 and SCF-2 as well as c-Kit. Corresponding results were obtained on immunocytochemical staining. Of three exemplary melanoma cell lines studied, two expressing SCF mRNA also released SCF spontaneously and on stimulation, whereas the line lacking SCF and c-kit mRNA (SK-Mel-23) failed to do so. These data demonstrate thus that melanoma cell lines, particularly those known to metastasize in vivo, lose the ability to express SCF-2 mRNA, suggesting that this molecule may serve, next to c-Kit, as a prognostic marker for malignant melanoma. © 2000 Cancer Research Campaign


*British Journal of Cancer* (2000) **82**(8), 1453–1458
© 2000 Cancer Research Campaign
DOI: 10.1054/ bjoc.1999.1076, available online at http://www.idealibrary.com on IDEAL®

### Expression of SCF splice variants in human melanocytes and melanoma cell lines: potential prognostic implications

**P Welker[1], D Schadendorf[3], M Artuc[1], J Grabbe[2] and BM Henz[1]**

[1]Department of Dermatology, Charité, Humboldt University, Augustenburger-Platz 1, D 13344 Berlin, Germany; [2]Medical University of Lübeck and [3]Clinical Cooperation Unit for Dermato-Oncology (DKFZ), Mannheim University Clinic, Germany

**Summary** Stem cell factor (SCF), the ligand for c-Kit, is known to regulate developmental and functional processes of haematopoietic stem cells, mast cells and melanocytes. Two different splice variants form predominantly soluble (sSCF or SCF-1) and in addition some membrane-bound SCF (mSCF or SCF-2). In order to explore the prognostic significance of these molecules in melanoma, total SCF, SCF splice variants and c-Kit expression were studied in normal skin melanocytes and in 11 different melanoma cell lines, using reverse transcription polymerase chain reaction, immunocytochemistry and enzyme-linked immunosorbent assay. Nine of the 11 melanoma cell lines expressed SCF-1 mRNA, only two of them SCF-2, and these two also SCF-1. Coexpression of both SCF-1 and c-Kit was noted in five cell lines, and only one cell line as well as normal melanocytes expressed both SCF-1 and SCF-2 as well as c-Kit. Corresponding results were obtained on immunocytochemical staining. Of three exemplary melanoma cell lines studied, two expressing SCF mRNA also released SCF spontaneously and on stimulation, whereas the line lacking SCF and c-kit mRNA (SK-Mel-23) failed to do so. These data demonstrate thus that melanoma cell lines, particularly those known to metastasize in vivo, lose the ability to express SCF-2 mRNA, suggesting that this molecule may serve, next to c-Kit, as a prognostic marker for malignant melanoma. © 2000 Cancer Research Campaign

**Keywords:** SCF splice variants; c-Kit; melanocytes; melanoma

Stem cell factor (SCF), also named mast cell growth factor or Steel factor, is the ligand for the tyrosine kinase receptor that is encoded by the c-Kit proto-oncogene (Zsebo et al, 1990). In c-Kit-expressing cells like mast cells and melanocytes, SCF plays a key role in several developmental and functional processes (Grabbe et al, 1994). Thus, SCF regulates induction of proliferation, differentiation, survival, integrin expression, migration and melanin pigmentation in melanoblasts (Miura et al, 1992; Lahav et al, 1994; Scott et al, 1994; Luo et al, 1995; Sviderskaya et al, 1995; Costa et al, 1996; Guo et al, 1997). Furthermore, SCF induces an increase of melanocyte numbers after subcutaneous injections (Grichnik et al, 1995; Costa et al, 1996).

SCF is produced by diverse cell types, including fibroblasts, keratinocytes and endothelial cells (Grabbe et al, 1994). A naturally occurring, soluble SCF form is released by extracellular proteolytic cleavage of the SCF protein at amino acid 165 near its transmembrane domain (SCF-1 or sSCF), possibly via the action of mast cell-specific chymase (Longley et al, 1997). An alternatively spliced mRNA lacking the codon for exon 6 and in turn encoding for amino acids that include the proteolytic cleavage site, induces translation of a functional SCF variant that tends to remain membrane-bound (SCF-2 or mSCF) (Flanagan et al, 1991; Huang et al 1992; Grabbe et al, 1994; Majumdar et al, 1994). The two SCF forms play distinct roles in the dispersal of normal melanocyte precursors, with SCF-1 being involved in the migration of the cells from the neural crest, whereas SCF-2 affects melanocyte survival and differentiation in the skin (Wehrle Haller et al, 1995). Using immunohistochemistry, Takahashi et al detected that few normal cutaneous melanocytes express SCF itself (Takahashi et al, 1995), and an SCF-dependent autocrine regulation of early melanogenesis has been discussed by Guo et al (1997). Expression of the splice variants has so far not been described in melanocytes, but both molecules have been detected in whole bone marrow, bone marrow stroma, peripheral blood neutrophils, human mast cells and HL-60 cell lines (Ramenghi et al, 1994; Welker et al, 1999). SCF as well as c-Kit have so far been demonstrated in various solid tumours and tumour cell lines (Turner et al, 1992; Cohen et al, 1994; Grabbe et al, 1994; Inoue et al, 1994), including malignant melanoma (Turner et al, 1992; Luo et al, 1995; Papadimitriou et al, 1995; Takahashi et al, 1995). In these studies, different cell types were found to vary widely in their ability to express either molecule. A potential prognostic significance of ligand or receptor have so far, however, only been explored for c-Kit. Thus, Natali et al (1992) reported that the progression of human cutaneous melanoma is associated with a loss of c-Kit expression. Furthermore, Huang et al (1996) observed that SCF induces increased retardation of melanoma growth, fewer lung metastases and increased apoptosis of melanoma cells overexpressing c-Kit. SCF–c-Kit interaction has also been shown to regulate melanocyte integrin expression (Scott et al, 1994), and integrins have recently been implicated in the process of melanoma metastasis (Schadendorf et al, 1993*a*, 1996). Expression of SCF-1 (sSCF) or SCF-2 (mSCF) by melanoma cells might thus also have prognostic implications.

*Received 9 December 1998*
*Revised 6 October 1999*
*Accepted 18 October 1999*

*Correspondence to:* P Welker

We have therefore investigated here the expression of c-Kit and both SCF-1 and SCF-2 in normal cutaneous melanocytes and in 11 different melanoma cell lines derived from primary melanomas or metastases at the protein and m-RNA level. Since the majority of the cell lines examined had previously been characterized regarding their metastatic potential in nude mice (Schadendorf et al, 1996), and since SCF-2-positive cell lines failed to metastasize in this model, SCF-2 expression may serve as a prognostic factor in melanoma.

### MATERIALS AND METHODS

### Cells

Cutaneous melanocytes, fibroblasts and keratinocytes were isolated from human foreskin specimens and cultured in special media allowing only growth of the respective cell type, as previously described (Grabbe et al, 1996). Melanocytes were kept in a special melanocyte growth medium (MGM-3, Bio-Whittaker, Walkersville, MD, USA) and made up 100% of cells after 1 week of culture, as determined with a melanocyte-specific antigen (TA99), (Dippel et al, 1995). Human melanoma cell lines WM 98-1 and WM 1341 were derived from primary melanomas (Herlyn et al, 1985, 1990; Lu et al, 1993) and were kindly provided by Dr M Herlyn (Wistar Institute, Philadelphia, PA, USA). MV3 human melanoma cells were established from a metastatic melanoma lymph node (van Muijen et al, 1991) and were kindly provided by Dr GN van Muijen (Nijmegen, The Netherlands). Several human melanoma cell lines were newly established and included two from cutaneous metastases (M7 and M13) (Dr U Morowietz, Kiel, Germany) (Schadendorf et al, 1996), one from a metastatic pleural effusion (UKRV-Mel-2), and one from a liver metastasis of a patient with an ocular melanoma (UKRV-Mel-4) (Artuc et al, 1995). SK-Mel-29 and MeWo, two human melanoma cell lines derived from metastatic lesions of patients with malignant melanoma, were kindly provided by the Memorial Sloan Kettering Cancer Center, New York, USA (a gift from Dr LJ Old). All cell lines were cultured in RPMI-1640 medium supplemented with 10% fetal calf serum, as described previously (Schadendorf et al, 1993*b*).

### Reverse transcription-polymerase chain reaction

Cells were lysed and total RNA prepared using the RNeasy-total-RNA-kit (Quiagen, Hilden, FRG). cDNA was synthesized by reverse transcription of 5 µg total RNA, using a cDNA synthesis kit (InVitrogen, Stade, USA). The following sets of oligonucleotide primers were used to amplify cDNA: **GAPDH:** 5′-GAT GAC ATC AAG AAG GTG GTG and 5′-GCT GTA GCC AAA TTC GTT GTC (197 bp) (Tokunaga et al, 1987), for **c-Kit**: 5′-CGT TGA CTA TCA GTT CAG CGA G and 5′-CTA GGA ATG TGT AAG TGC CTC (369 bp) (Ratajczak et al, 1992), **SCF$_{total}$** – primer-set I: 5′-GGG CTG GAT CGC AGC GC and 5′-CTC CAC AAG GTC ATC CAC **SCF$_{1+2}$** – primer set II: 5′-CTT CAA CAT TAA GTC CCT GAG and GTG TAG GCT GGA GTC TCC (Longley et al, 1993). **SCF-2** – primer set III: 5′-CTT GTG GAG TGC GTC AAA GA and 5′-TTG GCC TTC CCT TTC TCA GG. The expected fragment length was 276 bp for SCF primer-set I, 359 or 275 bp for SCF primer set II, dependent on the presence (SCF-1) or absence (SCF-2) of exon 6, and 199 bp for primer set III. The primer set III is specific for SCF-2 since it crosses the exon 5/7 boundary (10 bp of exon 5 and 10 bp of exon 7). Amplification was performed using *Taq* polymerase (Gibco) over 24–35 (40 cycles using primer set III) cycles with an automated thermal cycler (Perkin-Elmer, Weiterstadt, Germany). Each cycle consisted of the following steps: denaturation at 94°C, annealing at 58°C and extension at 72°C for 1 min each. For semiquantitative reverse transcription polymerase chain reaction (RT-PCR), cDNA from the different cell types was adjusted to equal quantities by serial dilutions, controlled by PCR amplification using primers of the housekeeping gene glyceraldehyde 3-phosphate dehydrogenase (GAPDH), as previously described (Grabbe et al, 1996). Linearity of the signal for GAPDH was determined between 24 and 27 cycles, and for the other markers between 30 and 35 cycles. PCR products were analysed by agarose gel electrophoresis using standard techniques, and staining intensity was quantitatively assessed by densitometric analysis with a video scanner (Biometra, Göttingen, Germany).

### Immunocytochemical staining

Immunocytochemistry was performed and evaluated on cytocentrifuge preparations of cells stained with the APAAP technique, as previously described (Hamann et al, 1994). Three different antibodies against SCF were used, a monoclonal antibody against SCF purchased from Genzyme (Cambridge, MA, USA), a mouse monoclonal antibody (HKL-12), and a rabbit polyclonal antibody (7278) (Hamann et al, 1995), both kindly donated by M Brockhaus, Basel, Switzerland. IgG1 was used as control and purchased from Dianova (Hamburg, Germany).

### Quantification of SCF protein

SCF in supernatants of cells seeded at $1 \times 10^6$ ml$^{-1}$ and cultured for 24 h in medium alone or stimulated with calcium ionophore A23187 (0.1 µM, Sigma, Deisenhofen, Germany) was measured using a commercial ELISA kit purchased from R&D Systems (Minneapolis, MN, USA). In addition, cells were incubated with 10 ng ml$^{-1}$ mouse-SCF (no cross reactivity in the ELISA kit), to displace SCF produced by melanocytes and potentially bound to its own SCF receptor on these cells.

### RESULTS

### SCF-specific m-RNA expression in melanocytes and melanoma cell lines

Evaluation of mRNA was done semiquantitatively by RT-PCR, using primer sets specific for c-Kit, SCF$_{total}$, and the splice variants SCF-1/SCF-2, or SCF-2 alone in comparison to GAPDH (Figure 1). The densitometric analysis of these data is shown in Table 1. Normal melanocytes were used as positive control (Figure 1) and yielded bands with the c-Kit and all SCF primers. Nine of the 11 melanoma lines also gave a positive signal with SCF primer set I which detects both splice variants of SCF with one signal (SCF$_{total}$) (Figure 1). With SCF primer set II which differentiates between the two splice variants, only two of these SCF-positive cell lines expressed SCF-2 (UKRV-Mel-4, WM 1341).

These results were confirmed by using a third primer set which is specific for the amplification of the SCF-2 splice variant with higher sensitivity. On RT-PCR analysis (Figure 1), a signal is evident in normal melanocytes and in the same two cell lines

 © 2000 Cancer Research Campaign

**Table 1** Densitometric data (signal intensity) of RT-PCR analysis (Figure 1) regarding c-kit and SCF mRNA expression in melanoma cell lines. All c-DNAs were normalized to the same level, using GAPDH as house-keeping gene

| Cell lines | c-kit | $SCF_{total}$ | SCF-1 | SCF-2 | Formation of metastases in nude mice[a] |
|---|---|---|---|---|---|
| MeWo | 450 | 300 | 180 | 0 | – |
| WM 98-1 | 0 | 80 | 20 | 0 | + |
| UKRV-Mel-4 | 380 | 410 | 230 | 100 | – |
| M13 | 30 | 10 | 30 | 0 | + |
| M7 | 0 | 300 | 100 | 0 | + |
| WM 1341 | 0 | 390 | 150 | 30 | – |
| UKRV-Mel-2 | 0 | 0 | 0 | 0 | + |
| SK-Mel-29 | 290 | 60 | 10 | 0 | ND |
| SK-Mel-23 | 0 | 0 | 0 | 0 | ND |
| SK-Mel-37 | 300 | 50 | 0 | 0 | ND |
| MV3 | 0 | 120 | 160 | 0 | + |

[a] Cell lines shown to induce metastases (+) or failing to do so (–) in nude mice (Schadendorf et al, 1996). ND – not done.

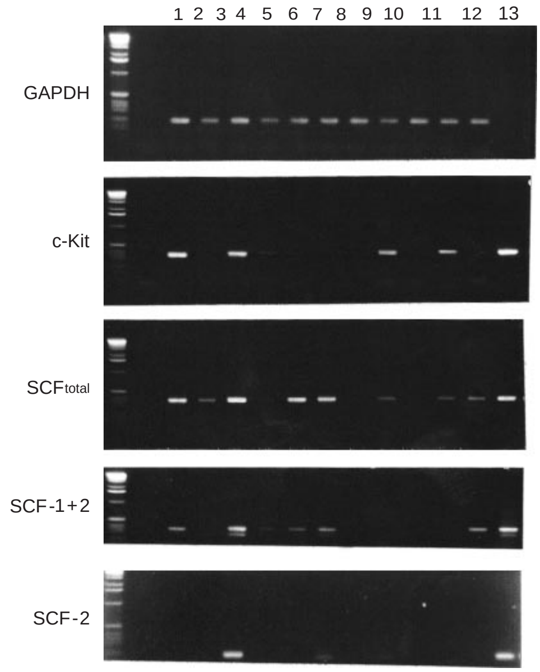

**Figure 1** Expression of m-RNA (semiquantitative RT-PCR) for GAPDH, c-Kit, $SCF_{total}$ (using primer set I) and SCF-1 and -2 (using primer set II to differentiate between full-length and spliced forms of SCF), and SCF-2 (using primer set III) (representative results from one of three experiments): 1: negative control (without c-DNA), 2: MeWo, 3: WM-98-1, 4: UKRV-Mel-4, 5: M13, 6: M7, 7: WM1341, 8: UKRV-Mel-2, 9: SK-Mel-29, 10: SK-Mel-23, 11: SK-Mel-37, 12: MV3, 13: normal human skin melanocytes (positive control)

which were positive using primer set II. In addition, a very rare signal is detectable in MeWo and SK-Mel-29. In only five of the SCF-positive cell lines, a signal was also found for c-Kit-specific m-RNA. Two cell lines failed to react with any of the primers studied, and four c-Kit-negative cell lines still produced $SCF_{total}$ (Table 1).

In a previous investigation (Schadendorf et al, 1996), the metastatic potential of eight of the 11 melanoma cell lines studied here was examined in vivo in a nude mouse model (Table 1). Interestingly, all five melanoma cell lines which were able to induce metastases in nude mice entirely lacked c-Kit or expressed it at a very low level. On the other hand, of the three tumours which had failed to induce metastases in vivo, two (MeWo and UKRV-Mel-4) were strongly positive for c-Kit, and the other two (UKRV-Mel-4 and WM1341) were the only cell lines expressing SCF-2. In the remaining cell lines, the signal intensity for SCF-1 failed to correlate with their ability to induce metastases.

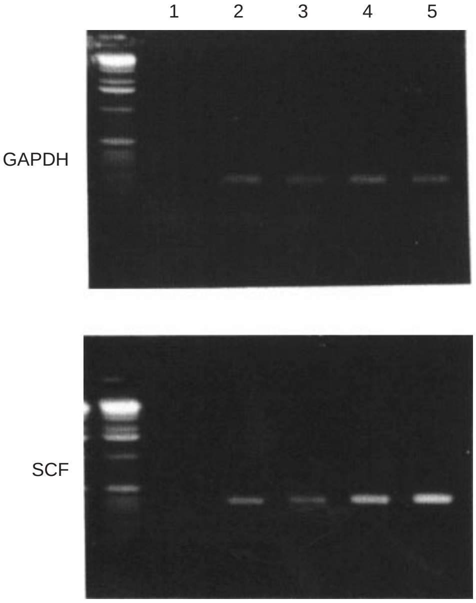

**Figure 2** Semiquantitative RT-PCR for GAPDH and SCF (primer set I) (representative results from one of three experiments): 1: negative. control (without c-DNA), 2: skin melanocytes, 3: MeWo, 4: skin fibroblasts, 5: skin keratinocytes

**Table 2** Release of SCF from unstimulated and stimulated melanoma cell lines

| Melanoma cell line | Incubation with Ca-ionophore[a] | Incubation with mouse SCF[a] | Release of SCF (pg/$10^6$ cells) |
|---|---|---|---|
| MEWO | – | – | $80 \pm 10$ |
| | – | + | $75 \pm 15$ |
| | + | – | $85 \pm 10$ |
| | + | + | $115 \pm 20$ |
| UKRV-Mel-4 | – | – | $55 \pm 5$ |
| | – | + | $55 \pm 10$ |
| | + | – | $70 \pm 10$ |
| | + | + | $60 \pm 10$ |
| SK-Mel-23 | – | – | 0 |
| | – | + | 0 |
| | + | – | 0 |
| | + | + | 0 |

(Means ± s.d. of $n = 3$ separate experiments) [a]for 24 h.

### Comparison of melanocyte SCF-expression with other SCF-producing cells

Besides melanocytes, several other cell types in the immediate vicinity of melanocytes and melanomas in the skin have also been

© 2000 Cancer Research Campaign

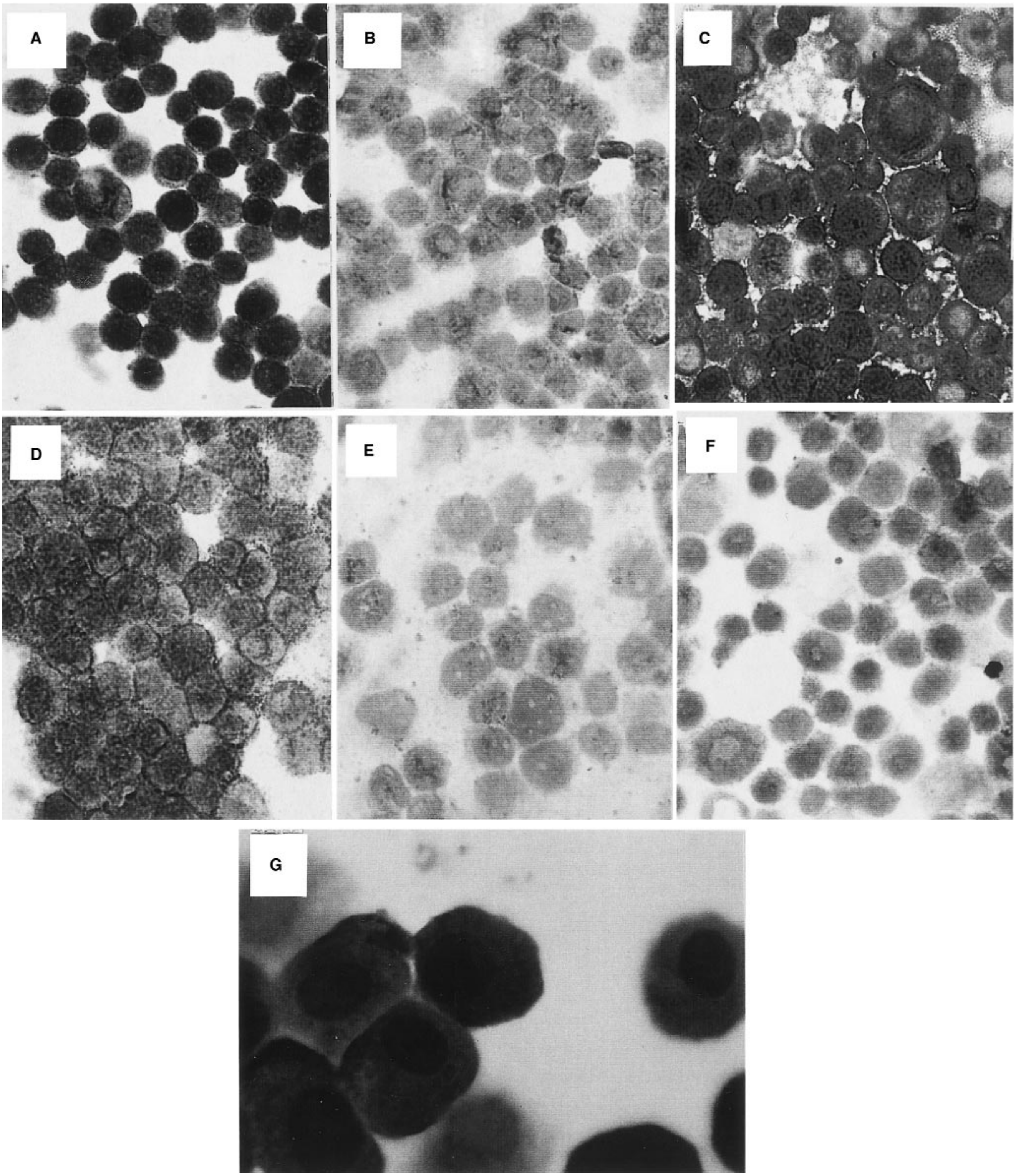

**Figure 3** Immunoreactivity of MeWo cells (**A**), WM 98-1 (**B**), UKRV-Mel-4 (**C**), WM 1341 (**D**), UKRV-Mel-2 (**E**), MV3 (**F**) and normal skin melanocytes (**G**) to a monoclonal antibody against SCF HKL-12 (APAAP technique, magnification: **A–F**: × 400; **G**: × 1000)

demonstrated to produce SCF. In order to assess the relative contribution of these cells compared to melanocytes regarding SCF expression, we studied the mRNA expression of $SCF_{total}$ in fibroblasts, keratinocytes, melanocytes and MeWo cells. This cell line was chosen because it expresses medium quantities of SCF, compared to the other melanoma cell lines. Figure 2 shows representative data of the semiquantitative RT-PCR for SCF expressed by the different cell types. On densitometric analysis, signal intensities were clearly lower for melanocytes and MeWo cells, compared to the other cell types, but they still ranged within the same order of magnitude (melanocytes: 120; MeWo: 85; fibroblasts: 310; keratinocytes 400).

 *© 2000 Cancer Research Campaign*

### Production and release of SCF protein in melanocytes

Analysis of the intensity of staining for SCF on immunocytochemistry (Figure 3) correlated well with the data obtained on semi-quantitative m-RNA analysis and held for all three different SCF antibodies tested. Interestingly, APAAP staining with the HKL-12 antibody exhibited cytoplasmatic as well as cell membrane SCF expression in the cell lines which had yielded an m-RNA signal for SCF-2 (UKRV-Mel-4, WM1341).

In order to examine whether these cells are also able to release SCF, we cultured three different cell lines (two positive and one negative for SCF on RT-PCR) for 24 h in serum-free culture medium alone or with Ca-ionophore A23187 added (Table 2). SCF protein was measurable in supernatants of the unstimulated and stimulated SCF-mRNA-positive cell lines (Table 1 and Figure 1) at approximately the same levels, whereas the SCF-mRNA-negative melanoma line released no measurable SCF under either condition. Since the first two melanoma cell lines studied also express the receptor for SCF and since part of the SCF secreted might be immediately bound to the receptor after release, mouse SCF was added to the cultures in order to prevent any binding of secreted SCF to its receptor (there is no cross-reactivity with mouse SCF on ELISA). As can be seen in Table 2, a substantial increase of measurable SCF was detected only in supernatants of ionophore-stimulated MeWo cells.

### DISCUSSION

In the present study, melanocytes are shown for the first time to express both splice variants of SCF, namely SCF-1 and SCF-2. Since they also express c-Kit, cutaneous melanocytes are like mast cells potentially subject to autocrine regulation, in contrast to neutrophils and HL-60 cells which have been reported to express both splice variants of SCF, but not c-Kit (Ramenghi et al, 1994; Welker et al, 1999).

In contrast to melanocytes, the expression of these molecules is partially and variably lost in melanoma cell lines, particularly with regard to SCF-2. If present, SCF expression is, however, comparable to that in melanocytes and lower compared to keratinocytes and fibroblasts. These latter can thus potentially exert control over normal growth and differentiation of melanocytes in the basal layer of the epidermis.

So far, differentiation between the SCF-splice variants in melanoma lines has been reported only for the HT144 cells which also express c-Kit (Turner et al, 1992). SCF mRNA has been described in all five different melanoma lines studied by another group, although the splice variants were not distinguished (Papadimitriou et al, 1995). Concurrent expression of c-Kit and SCF-mRNA was found on only three of these five cell lines, and c-Kit protein even in only one of them, namely in MeWo cells which were also included in the present study. Another group detected SCF protein in only one of five melanoma cell lines, none of which expressed c-Kit, whereas benign melanocytic naevi expressed both molecules (Takahashi et al, 1995). In the present study, c-Kit mRNA was identified in five of the 11 cell lines, in agreement with a previous report from our group regarding c-Kit protein expression in melanoma cell lines (Worm et al, 1993). Interestingly, immunohistochemical studies demonstrated that melanocytic naevus cells express c-Kit only in the epidermal and not in the dermal compartment (Grabbe et al, 1994; Takahashi et al, 1995). Dermal melanocytic naevus cells also lack pigmentation, suggesting that they are no longer subject to SCF-dependent melanogenesis. Taken together, these observations suggest that melanocytic cells lose the ability to express SCF and c-Kit, possibly in dependence of their life cycle and/or in response to their microenvironment.

There are a number of indications that SCF control over the cell might regulate tumour development and spread. Thus, although the factor has no major effects on growth of different cell lines (Papadimitriou et al, 1995), it has been reported that an enforced overexpression of c-Kit renders melanoma cells susceptible to stem cell factor-induced apoptosis and inhibits the tumorigenic and metastatic potential of the cells (Huang et al, 1996). The degree of SCF expression could, however, not be correlated with metastatic potential until now. The present data, showing that the splice variant which encodes for the mainly membrane-bound SCF-2 and not the total SCF-specific m-RNA is important, shed new light on this question. Thus, only those melanoma cell lines which express neither c-Kit m-RNA (cell line M13 minimal amounts) nor SCF-2 m-RNA have in the past been shown to induce metastases in the nude mice model (Table 1).

Previous studies have shown that membrane bound forms of various growth factors, like SCF, are able to interact with their respective receptor expressed by a neighbouring target cell (juxtacrine interaction) (Bosenberg et al, 1993). This membrane-bound factor which is biologically as active as a freely diffusible factor, may similarly mediate specific cell-to-cell contacts, enhancing differentiation and survival of neighbouring melanocytes. Possibly, c-Kit and membrane-bound SCF-2 is also of importance on interaction of melanocytic cells and mast cells, the only other skin cells expressing c-Kit-like melanocytes. Mast cells are located primarily in the upper dermis, only rarely in the epidermis, and they have been shown to increase in number and infiltrate dermal melanomas (Schadendorf et al, 1995). The potential functional significance of mast cells in melanomas is, however, as yet unclear, as is their prognostic significance, although direct interaction with melanoma cells via SCF-2/c-Kit is well possible. Mast cell infiltration may on the other hand be induced, next to anaphylatoxins, by chemotactically active SCF-1 released from melanoma cells as well as surrounding endothelial cells, fibroblasts and keratinocytes (Grabbe et al, 1994; Hartmann et al, 1998).

Taken together, soluble and membrane-bound SCF may function in an autocrine and paracrine fashion, modulating melanin synthesis and apoptosis of melanocytic cells and potentially also their tumorigenic potential. The relative prognostic significance of the splice variants of SCF and c-Kit in melanoma, and particularly of SCF-2 which is strikingly rare in melanoma cell lines, as observed here, will have to be further confirmed in studies of primary and metastatic melanoma, placing its expression in relationship to patients' prognosis.

### ACKNOWLEDGEMENTS

This work was supported by grants He 2686/8-2 and He2686/8-3 from the German Research Foundation (DFG). The authors wish to thank Mrs Regina Nordheim and Mrs Elke Bauer for excellent technical assistance.

### REFERENCES

Artuc M, Nürnberg W, Czarnetzki BM and Schadendorf D (1995) Characterization of gene regulatory elements for selective gene expression in human melanoma cells. *Biochem Biophys Res Commun* **213**: 699–705

© 2000 Cancer Research Campaign 

Bosenberg MW and Massague J (1993) Juxtacrine cell signaling molecules. *Curr Opin Cell Biol* **5**: 832–838

Cohen PS, Chan JP, Lipkunskaya M, Biedler JL and Seeger RC (1994) Expression of stem cell factor and c-kit in human neuroblastoma. The Children's Cancer Group. *Blood* **84**: 3465–3472

Costa JJ, Demetri GD, Harrist TJ, Dvorak AM, Hayes DF, Merica EA, Menchaca DM, Gringeri AJ, Schwartz LB and Galli SJ (1996) Recombinant human stem cell factor (kit ligand) promotes human mast cell and melanocyte hyperplasia and functional activation in vivo. *J Exp Med* **183**: 2681–2686

Dippel E, Haas N, Grabbe J, Schadendorf D, Hamann K and Czarnetzki BM (1995) Expression of the c-kit receptor in hypomelanosis: a comparative study between piebaldism, naevus depigmentosus and vitiligo. *Br J Dermatol* **132**: 182–189

Flanagan JG, Chan DC and Leder P (1991) Transmembrane form of the kit ligand growth factor is determined by alternative splicing and is missing in the Sld mutant. *Cell* **64**: 1025–1035

Grabbe J, Welker P, Dippel E and Czarnetzki BM (1994) Stem cell factor, a novel cutaneous growth factor for mast cells and melanocytes. *Arch Dermatol Res* **287**: 78–84

Grabbe J, Welker P, Rosenbach T, Nürnberg W, Krüger-Krasagakes S, Artuc M, Fiebiger E and Henz BM (1996) Release of stem cell factor (SCF) from human keratinocytes (HaCaT) is increased in differentiating versus proliferating cells. *J Invest Dermatol* **107**: 219–224

Grichnik JM, Crawford J, Jimenez F, Kurtzberg J, Buchanan M, Blackwell S, Clark RE and Hitchcock MG (1995) Human recombinant stem-cell factor induces melanocytic hyperplasia in susceptible patients. *J Am Acad Dermatol* **33**: 577–583

Guo CS, Wehrle-Haller B, Rossi J and Ciment G (1997) Autocrine regulation of neural crest cell development by Steel factor. *Dev Biol* **184**: 61–69

Hamann K, Grabbe J, Welker P, Haas N, Algermissen B, Czarnetzki BM (1994) Phenotypic evaluation of cultured human mast and basophilic cells and of normal human skin mast cells. *Arch Dermatol Res* **286**: 380–385

Hamann K, Haas N, Grabbe J and Czarnetzki BM (1995) Expression of stem cell factor in cutaneous mastocytosis. *Br J Dermatol* **133**: 203–208

Hartmann K, Henz BM, Krüger-Krasagakes S, Köhl J, Burger R, Guhl S, Haase I, Lippert U and Zuberbier T (1997) C3a and C5a stimulate chemotaxis of human mast cells. *Blood* **89**: 2863–2870

Herlyn M (1990) Human melanoma: development and progression. *Cancer Metastasis Rev* **9**: 101–112

Herlyn M, Thurin J, Balaban G, Bennicelli JL, Herlyn D, Elder DE, Bondi E, Guerry D, Nowel P and Clark WH (1985) Characteristics of cultured human melanocytes isolated from different stages of tumor progression. *Cancer Res* **45**: 5670–5476

Huang EJ, Nocka KH, Buck J and Besmer P (1992) Differential expression and processing of two cell associated forms of the kit-ligand: KL-1 and KL-2. *Mol Biol Cell* **3**: 349–362

Huang S, Luca M, Gutman M, McConkey DJ, Langley KE, Lyman SD and Bar Eli M (1996) Enforced c-KIT expression renders highly metastatic human melanoma cells susceptible to stem cell factor-induced apoptosis and inhibits their tumorigenic and metastatic potential. *Oncogene* **13**: 2339–2347

Inoue M, Kyo S, Fujita M, Enomoto T and Kondoh G (1994) Coexpression of the c-kit receptor and the stem cell factor in gynecological tumors. *Cancer Res* **54**: 3034–3053

Lahav R, Lecoin L, Ziller C, Nataf V, Carnahan JF, Martin FH and Le Douarin NM (1994) Effect of the Steel gene product on melanogenesis in avian neural crest cell cultures. *Differentiation* **58**: 133–139

Longley BJ Jr, Morganroth GS, Tyrrell L, Ding TG, Anderson DM, Williams DE and Halaban R (1993) Altered metabolism of mast-cell growth factor (c-kit ligand) in cutaneous mastocytosis. *N Engl J Med* **328**: 1302–1307

Longley BJ, Tyrrell L, Ma Y, Williams DA, Halaban R, Langley K, Lu HS and Schechter NM (1997) Chymase cleavage of stem cell factor yields a bioactive, soluble product. *Proc Nat Acad Sci USA* **94**: 9017–9021

Lu C and Kerbel RS (1993) Interleukin-6 undergoes transition from paracrine growth inhibitor to autocrine stimulator during human melanoma progression. *J Cell Biol* **120**: 1281–1288

Luo D, Chen H, Searles G and Jimbow K (1995) Coordinated mRNA expression of c-Kit with tyrosinase and TRP-1 in melanin pigmentation of normal and malignant human melanocytes and transient activation of tyrosinase by Kit/SCF-R. *Melanoma Res* **5**: 303–309

Majumdar MK, Feng L, Medlock E, Toksoz D and Williams DA (1994) Identification and mutation of primary and secondary proteolytic cleavage sites in murine stem cell factor cDNA yields biologically active, cell-associated protein. *J Biol Chem* **269**: 1237–1242

Miura N and Suda T (1992) Stem cell factor/c-kit interaction in primordial germ cell, melanoblast and hematopoietic progenitors. *Gan To Kagaku Ryoho* **19**: 1777–1785

Natali PG, Nicotra MR, Winkler AB, Cavaliere R, Bigott A and Ullrich A (1992) Progression of human cutaneous melanoma is associated with loss of expression of c-kit proto-oncogene receptor. *Int J Cancer* **52**: 197–201

Papadimitriou CA, Topp MS, Serve H, Oelmann E, Koenigsmann M, Meurer J, Oberberg D, Reufi B, Thiel E and Berdel WE (1995) Recombinant human stem cell factor does exert minor stimulation of growth in small lung cancer and melanoma cell lines. *Eur J Cancer Part A: Gen Top* **31**: 2371–2378

Ramenghi U, Ruggieri L, Dianzani I, Rosso C, Brizzi MF, Camashcella C, Pietsch T and Saglio G (1994) Human peripheral blood granulocytes and myeloid cell lines express both transcripts encoding for stem cell factor. *Stem Cells* **12**: 521–526

Ratajczak MZ, Luger SM, DeRiel K, Abrahm J, Calabretta B and Gewirtz AM (1992) Role of the KIT protooncogene in normal and malignant human hematopoiesis. *Proc Natl Acad Sci USA* **89**: 1710–1714

Schadendorf D, Gawlik C, Haney U, Ostmeier H, Suter L and Czarnetzki BM (1993*a*) Tumor progression and metastatic behavior in vivo correlates with integrin expression on melanocytic tumors. *J Pathol* **170**: 429–434

Schadendorf D, Möller A, Algermissen B, Worm M, Sticherling M and Czarnetzki BM (1993*b*) IL-8 produced by human malignant melanoma cells in vitro is an essential autocrine growth factor. *J Immunol* **151**: 2667–2675

Schadendorf D, Kohlmus C, Gawlik C, Suter L and Czarnetzki BM (1995) Mast cells in melanocytic tumours. *Arch Dermatol Res* **287**: 452–456

Schadendorf D, Fichtner I, Makki A, Alijagic S, Kupper M, Mrowietz U and Henz BM (1996) Metastatic potential of human melanoma cells in nude mice – characterisation of phenotype, cytokine secretion and tumour-associated antigens. *Br J Cancer* **74**: 194–199

Scott G, Ewing J, Ryan D and Abboud C (1994) Stem cell factor regulates human melanocyte–matrix interactions. *Pigment Cell Res* **7**: 44–51

Sviderskaya EV, Wakeling WF and Bennett DC (1995) A cloned, immortal line of murine melanoblasts inducible to differentiate to melanocytes. *Development* **121**: 1547–1557

Takahashi H, Saitoh K, Kishi H and Parsons PG (1995) Immunohistochemical localisation of stem cell factor (SCF) with comparison of its receptor c-Kit proto-oncogene product (c-KIT) in melanocytic tumours. *Virchows Arch* **427**: 283–288

Tokunaga K, Nakamura Y, Sakata K, Fujimori K, Ohkubo M, Sawada K and Sakiyama S (1987) Enhanced expression of a glyceraldehyde-3-phosphate dehydrogenase gene in human lung cancers. *Cancer Res* **47**: 5616–5619

Turner AM, Zsebo KM, Martin F, Jacobsen FW, Bennett LG and Broudy VC (1992) Nonhematopoietic tumor cell lines express stem cell factor and display c-kit receptors. *Blood* **80**: 374–381

Van Muijen GN, Jansen KF, Cornelissen IM, Smeets DF, Beck JL and Ruiter DJ (1991) Establishment and characterization of a human melanoma cell line (MV3) which is highly metastatic in nude mice. *Int J Cancer* **48**: 85–91

Wehrle Haller B and Weston JA (1995) Soluble and cell-bound forms of steel factor activity play distinct roles in melanocyte precursor dispersal and survival on the lateral neural crest migration pathway. *Development* **121**: 731–742

Welker P, Grabbe J and Henz BM (1999) Human mast cells produce and differentially express soluble and membrane-bound stem cell factor. *Scand J Immunol* **49**: 495–500

Worm M, Reichert U, Czarnetzki BM and Schadendorf D (1993) Expression of growth factor receptors on human melanoma cells: comparison of modulating effects of interferons and retinoids. *Exp Dermatol* **2**: 217–223

Zsebo KM, Williams DA, Geissler EN, Broudy VC, Martin FH, Atkins HL, Hsu RY, Birkett NC, Okino KH, Murdock DC, Jacobson FW, Langley KE, Smith KA, Takeishi T, Cattenac BM, Galli SJ and Suggs SV (1990) Stem cell factor is encoded at the Sl locus of the mouse and is the ligand for the c-kit tyrosine kinase receptor. *Cell* **63**: 213–224

 *© 2000 Cancer Research Campaign*
